# Population- and genome-specific patterns of linkage disequilibrium and SNP variation in spring and winter wheat (*Triticum aestivum *L.)

**DOI:** 10.1186/1471-2164-11-727

**Published:** 2010-12-29

**Authors:** Shiaoman Chao, Jorge Dubcovsky, Jan Dvorak, Ming-Cheng Luo, Stephen P Baenziger, Rustam Matnyazov, Dale R Clark, Luther E Talbert, James A Anderson, Susanne Dreisigacker, Karl Glover, Jianli Chen, Kim Campbell, Phil L Bruckner, Jackie C Rudd, Scott Haley, Brett F Carver, Sid Perry, Mark E Sorrells, Eduard D Akhunov

**Affiliations:** 1 USDA ARS Genotyping Laboratory, Biosciences Research Laboratory, Fargo, ND, USA; 2 Department of Plant Sciences, University of California, Davis, CA, USA; 3 Plant Science Building, University of Nebraska, Lincoln, NE, USA; 4 Department of Plant Pathology, Kansas State University, Manhattan, KS, USA; 5 WestBred, LLC, Bozeman, MT, USA; 6 Department of Plant Sciences, Montana State University, Bozeman, MT, USA; 7 Dept. of Agronomy & Plant Genetics, University of Minnesota, St. Paul, MN, USA; 8 Genetic Resources and Enhancement Unit, CIMMYT, Mexico, D.F., Mexico; 9 Plant Science Department, South Dakota State University, Brookings, SD, USA; 10 University of Idaho Aberdeen Research & Extension Center, Aberdeen ID, USA; 11 USDA-ARS Wheat Genetics, Quality, Physiology & Disease Research Unit, Washington State University, Pullman WA, USA; 12 Plant Sciences and Plant Pathology, Bozeman, MT, USA; 13 Texas AgriLife Research and Extension Center, Amarillo, TX, USA; 14 Soil and Crop Sciences Department, Colorado State University, Fort Collins, CO, USA; 15 Oklahoma State University, Department of Plant and Soil Sciences, Stillwater, OK, USA; 16 WestBred, LLC, Haven, KS, USA; 17 Plant Breeding and Genetics, Cornell University, Ithaca, NY, USA; 18 Institute of Biochemistry and Genetics, RAS, Ufa Russia

## Abstract

**Background:**

Single nucleotide polymorphisms (SNPs) are ideally suited for the construction of high-resolution genetic maps, studying population evolutionary history and performing genome-wide association mapping experiments. Here, we used a genome-wide set of 1536 SNPs to study linkage disequilibrium (LD) and population structure in a panel of 478 spring and winter wheat cultivars (*Triticum aestivum*) from 17 populations across the United States and Mexico.

**Results:**

Most of the wheat oligo pool assay (OPA) SNPs that were polymorphic within the complete set of 478 cultivars were also polymorphic in all subpopulations. Higher levels of genetic differentiation were observed among wheat lines within populations than among populations. A total of nine genetically distinct clusters were identified, suggesting that some of the pre-defined populations shared significant proportion of genetic ancestry. Estimates of population structure (F_ST_) at individual loci showed a high level of heterogeneity across the genome. In addition, seven genomic regions with elevated F_ST _were detected between the spring and winter wheat populations. Some of these regions overlapped with previously mapped flowering time QTL. Across all populations, the highest extent of significant LD was observed in the wheat D-genome, followed by lower LD in the A- and B-genomes. The differences in the extent of LD among populations and genomes were mostly driven by differences in long-range LD ( > 10 cM).

**Conclusions:**

Genome- and population-specific patterns of genetic differentiation and LD were discovered in the populations of wheat cultivars from different geographic regions. Our study demonstrated that the estimates of population structure between spring and winter wheat lines can identify genomic regions harboring candidate genes involved in the regulation of growth habit. Variation in LD suggests that breeding and selection had a different impact on each wheat genome both within and among populations. The higher extent of LD in the wheat D-genome versus the A- and B-genomes likely reflects the episodes of recent introgression and population bottleneck accompanying the origin of hexaploid wheat. The assessment of LD and population structure in this assembled panel of diverse lines provides critical information for the development of genetic resources for genome-wide association mapping of agronomically important traits in wheat.

## Background

In crops, the level of genetic diversity and linkage disequilibrium (LD) can be affected by various factors including demography and inbreeding [1-6], selection for favorable alleles [7,8], domestication [2,9,10], outcrossing of crop cultivars with genetically distinct lines of wild ancestors and landraces [1,11,12] and admixture [13,14]. Genetic diversity of domesticated crops is usually reduced compared to wild ancestors [2,6,9,15,16]. In tetraploid wheat, the population bottleneck that accompanied tetraploid emmer wheat domestication about 10,000 years ago [17] reduced nucleotide diversity by 30 to 50% in the A- and B-genomes, depending on the study and diversity measure used [15,18]. Diversity was further reduced in hexaploid wheat as a consequence of the polyploidy bottleneck resulting from hexaploid wheat speciation [18,19]. Different rates of gene flow from the ancestors of hexaploid wheat, tetraploid wheat for the A- and B-genomes and *Aegilops tauschii *for the D-genome [20,21] resulted in different levels of diversity in hexaploid wheat genomes [19]. While diversity levels are similar in the A- and B-genomes, it is greatly reduced in the D-genome [18,19]. The D-genome also shows higher levels of LD than the A- and B-genomes [19,22].

Interpreting patterns of genetic diversity in modern crop cultivars is further complicated by strong selection and interbreeding with landraces and genetically distant wild relatives. Regions of the genome subjected to recent selection or introgressions from landraces or wild relatives were shown to have elevated LD and low genetic diversity [7,12,23]. Factors such as inbreeding, human- and environment-driven selection, founder effect and gene flow influence the distribution of genetic variation both across the genome and between populations resulting in the formation of genetically differentiated groups [2-6]. Strong population structure has been reported for many crops including wheat [5,22,24,25]. A thorough understanding of population structure has important implications as population structure is one of the major reasons for false associations between phenotypes and markers in association mapping (AM) studies [26]. Hence, the inclusion of population structure estimates in AM is important for reducing spurious associations [27].

On the practical level, the distribution of genetic diversity in modern cultivars plays an important role in the choice of specific mapping and crop improvement strategies. In recent years, association mapping was shown to be a powerful method for complementing the traditional gene mapping studies based on controlled crosses [5,28-30]. The extent of LD defines the marker density required for genome-wide association mapping (GWAM). GWAM experiments in human and natural plant populations require several hundred thousand SNPs for finding a marker allele linked to a causal mutation [4,31]. However, the elevated level of LD in crop populations suggests that a smaller number of markers can provide sufficient genome coverage for finding marker-trait associations. Indeed, in two-row spring barley cultivars significant intra-chromosomal LD extended up to 15 cM [3]. Analysis of LD patterns in U.S. wheat populations showed significant LD extended to 5 cM [25] or 10 cM [22] while some populations of durum wheat (*T. turgidum*) retained more than 50% of their initial LD value at distances up to 20 cM [24]. In theory, by selecting a set of closely related cultivars it should be possible to increase the extent of LD and use fewer markers for detecting associations.

Recent advances in DNA sequencing and genotyping have enabled genome-wide studies capable of characterizing genetic variation and the extent of LD in natural and breeding populations. Single nucleotide polymorphism (SNP) has become the most frequently used type of molecular marker for these analyses in many species because of their high abundance across the genome and the availability of cost-effective high-throughput genotyping assays [32-34]. One of the first sets of SNPs developed for polyploid wheat [19] was used in this study to design a 1536-plex wheat oligo pool assay (wheat OPA) to analyze the patterns of SNP variation and LD in diverse populations of cultivated spring and winter wheat lines from the US and CIMMYT ("Centro Internacional de Mejoramiento de Maíz y Trigo") breeding programs. This knowledge is critical to the design of valid GWAM experiments in wheat and useful for understanding the role of selection and breeding in the distribution of genetic variation across the wheat genome.

## Methods

### Plant material

The wheat lines included in our study represent diverse cultivars utilized in 17 wheat breeding programs including 9 winter and 8 spring wheat populations. All cultivars were selected to represent the current genetic and phenotypic diversity of a specific state's breeding program. The phenotypic traits targeted by breeding programs include: disease resistance (leaf, stripe and stem rusts), winter survival for winter wheat breeding programs in the Midwest, end-use quality, terminal heat tolerance, resistance to drought stress, yield potential, early maturity, resistance to the wheat stem sawfly and herbicide tolerance. The complete list of lines with their pedigrees is provided in the Additional File 1.xls. Plants were grown in a greenhouse and DNA was extracted from the leaves of 4-6 week old seedlings using methods described before [35].

### SNP genotyping

The SNPs discovered in a panel of 32 lines of tetraploid and hexaploid wheat were downloaded from the Wheat SNP Database [36]. SNP selection and assay design were performed according to previously described procedures [32]. The following criteria were applied for SNP selection: no more than 2 SNPs were selected per locus, with preference being given to SNPs present in at least two lines in the discovery panel. Additional SNPs were discovered by sequencing the transcriptomes of *T. aestivum *cv. Chinese Spring and Jagger. Repetitive elements were detected and masked by comparing sequences with the TREP [37] and GIRI [38] databases. The masked sequences were submitted to Illumina for processing by Illumina^® ^Assay Design Tool (ADT). The ADT generates designability rank scores for each SNP that can vary from 0 to 1. The SNPs with scores above 0.6 have a high probability of being converted into a successful genotyping assay. A total of 1536 SNPs were selected for developing the wheat OPA (Additional File 2.xls). Genotyping was performed at the USDA-ARS genotyping laboratory in Fargo, North Dakota according to standard Illumina GoldenGate assay protocols [39]. Subsequent genotype calling was carried out using Illumina's BeadStudio software v.3. The accuracy of the genotype call was manually evaluated for the misclassification of homozygous and heterozygous clusters using the software's clustering algorithm. This step proved critical for reducing the genotyping error rate associated with peculiarities of clustering patterns in polyploid wheat. Following the removal of loci with low-quality clustering, the previously estimated genotyping error rate for hexaploid wheat was a mere 1% [32].

### Genetic diversity

Genetic diversity was evaluated by calculating the polymorphism information content ($PIC=1-\sum_{i}^{n}p_{i}^{2}$, where *p_i _*is the frequency of the *i*-th allele [40]) for the number of alleles across and within breeding programs using PowerMarker software [41]. Analyses were performed separately on four datasets: three datasets included SNPs grouped by genome and one dataset included complete set of SNPs.

### Population structure

For analysis of population structure, the SNP dataset was divided into the three genome-specific datasets and one combined dataset. To reduce the effect of frequency correlation between linked alleles, we selected SNP loci located approximately 4 cM or farther apart from each other. The A-genome dataset included 91 SNP loci while the B-genome and D-genome dataset included 89 and 39 SNP loci, respectively (Additional File 3.xls). We assumed that each individual in the population was homozygous for all loci, and heterozygous loci were treated as missing data. The proportion of heterozygous loci in our dataset was 0.5%. The population structure was inferred using the model-based Bayesian clustering approach implemented in the program Structure [42]. A total of 10 iterations of Gibbs sampler were run for an admixture model with both correlated and non-correlated allele frequencies [43]. Lengths of burn-in and simulation runs of 10^5 ^and 10^6^, respectively, were selected based on the convergence of summary statistics (log probability of data) among several independent runs. Results of independent runs for the same value of K were summarized using the CLUMPP program [44].

The number of populations (K) present in the dataset was estimated by plotting the probability of data ln Pr(X|K) for each value of K. The variation of ln Pr (X|K) among independent simulation runs with the same value of K and the rate of ln Pr (X|K) change from K-1 to K was used to select the optimal number of populations in the sample (Additional File 4.ppt). Two *ad hoc *methods for estimating the correct number of K in the sample, suggested by Pritchard et al. [42] and Evanno et al. [45], were also tested. Population assignments for each individual were visualized using Distruct software [46]. Similar analyses were performed using the program InStruct [47]. This program extends the Bayesian clustering algorithm implemented in the program Structure [42] by removing the assumption of Hardy-Weinberg disequilibrium within inferred clusters and relying instead on selfing rates to calculate expected genotype frequencies [47]. The simulations were run for 100,000 steps after 50,000 burn-in iterations.

Population structure was also analyzed using the principal component analysis (PCA) implemented in the software EIGENSTRAT [48]. Data from 597 polymorphic SNPs with minor allele frequency >0.05 was used to assess the clustering of genetic variation among all 478 samples investigated.

The differentiation of populations was further investigated by estimating F_ST _for individual loci and the components of variance for two levels of population hierarchy using methods described by Weir and Cockerham [49] and Weir [50] as implemented in the software package PowerMarker [39]. F_ST _provides a measure of population differentiation by estimating the correlation of alleles within the same sub-population relative to that found in the entire population. The overall distribution of genetic variation in the wheat cultivars was estimated for two levels of population hierarchy: growth habit (spring and winter wheat) and origin (breeding populations). The winter wheat population from Kansas was excluded from the analysis due to the insufficient number of lines. The mean F_ST _values in a sliding window of 5 consecutive linked SNPs were calculated to identify genomic regions genetically differentiated between spring and winter wheat lines. A 95% confidence interval (CI) for mean F_ST _values was estimated by sampling 1,000 times with replacement the sets of 5 SNPs randomly selected from the 849-SNP dataset and taking the 95^th ^percentile of the distribution of means.

Regions of the wheat genome showing elevated F_ST _levels were compared with the positions of previously mapped or cloned flowering time QTL. The sequences of genes containing SNPs included in the wheat OPA were compared with the sequences of gene-derived flanking molecular markers (ESTs, cDNA) used in QTL mapping studies. The syntenic relationship between wheat, rice and Brachypodium genomes was used to compare and validate map positions.

### Linkage disequilibrium

For measuring LD, the locations of gene loci harboring SNPs on the *Ae. tauschii *genetic map reported by Luo et al. [51] were used. Pair-wise linkage disequilibrium (LD) was measured using the squared allele-frequency correlations, *r*^2 ^, according to Weir [50]. In order to reduce the variation of LD estimates generated by the inclusion of rare alleles, only SNP alleles with minor allele frequency (MAF) higher than 0.05 were used in these calculations. LD levels and the rate of LD decay were assessed by calculating *r*^2 ^for pairs of SNP loci and plotting them against genetic distance. The relationship between LD decay and genetic distance was summarized by fitting a locally-weighted linear regression (loess) line to *r*^2 ^data. The statistical significance of individual *r*^2 ^estimates was calculated by the exact test following Weir [50]. The false discovery rate (FDR) was established at 0.01 using the Benjamini & Hochberg method [52]. Chromosome specific *r*^2 ^values were plotted using the R package LDheatmap [53]. Blocks of SNPs showing elevated levels of LD were identified using the method described by Gabriel et al. [54] and implemented in the program Haploview [55]. Background LD was estimated as the 95^th^-percentile of the distribution of *r*^2 ^values for unlinked SNP loci [25].

## Results

### SNP genotyping and variation

The genotyping of 478 spring and winter wheat lines with multiplexed 1,536 Illumina Golden Gate SNP assay generated 734,208 genotyping data points (Table 1). After the removal of SNPs failing to generate clear genotype clustering, 1,299 SNPs with high quality genotype calls were obtained with a 85% success of SNP conversion into the working genotyping assays. Considering these SNPS, 849 were polymorphic among the 478 lines included in this study. Most genotypes were homozygous (400,328 = 98.6%) with only a small fraction showing residual heterozygocity (1,961 = 0.5%) or no amplification (3,533 = 0.9%). Eighty-three percent of the SNPs were polymorphic in both spring and winter wheat populations. Among the 849 polymorphic SNPs, only 52 and 97 SNPs were monomorphic in the panels of 241 spring and 237 winter wheat lines, respectively (Table 2). A high proportion of polymorphic SNPs (70% - 85%) was recovered within populations of different origin, suggesting a high level of diversity within all U.S. breeding populations (Table 2). After exclusion of two winter wheat populations (KS and NY) due to their relatively small sample sizes (4 and 10, respectively), the estimates of polymorphism level (PIC) varied within a very narrow range, between 0.14 to 0.16 among the winter populations and from 0.14 to 0.18 among the spring wheat populations.

**Table 1:** Wheat OPA evaluation

Genome	Total assayed	No. failed SNPs	No. good SNPs	No. polymorphic SNPs (%)	No. alleles detected	PIC
A	642	93	549	368 (67%)	1.67	0.165
B	675	109	566	374 (66%)	1.67	0.170
D	219	35	184	107 (58%)	1.60	0.120
Total	1536	237	1299	849 (65%)		

Mean					1.66	0.160

**Table 2:** Average estimates of minor allele frequency, number of alleles per locus, and polymorphism information content.

Population	Growth Habit	Origin	Number of lines	Proportion of polymorphic SNPs, (%)	MAF	Number of alleles	PIC
SD	winter	SD	21	79.5	0.13	1.62	0.14
NE	winter	NE	49	84.8	0.12	1.7	0.14
OK	winter	OK	40	84.8	0.14	1.7	0.16
WB	winter	WestBred	11	78.4	0.15	1.59	0.16
CO	winter	CO	30	82.7	0.13	1.64	0.15
TX	winter	TX	38	85	0.14	1.73	0.16
MT	winter	MT	34	82.6	0.12	1.65	0.14
KS	winter	KS	4	64.9	0.1	1.32	0.1
NY	winter	NY	10	70.3	0.1	1.44	0.11

Winter wheat	winter		237	89.2	0.14	1.89	0.17

WB	spring	WestBred	30	82.4	0.14	1.66	0.16
SD	spring	SD	40	80.7	0.13	1.6	0.14
MT	spring	MT	30	85	0.17	1.71	0.18
MN	spring	MN	40	84.8	0.14	1.71	0.16
ID	spring	ID	30	83.2	0.16	1.65	0.17
WA	spring	WA	10	78.1	0.15	1.56	0.16
CA	spring	CA	30	87	0.16	1.72	0.18
CM	spring	CIMMYT	31	85.2	0.14	1.72	0.16

Spring wheat	spring		241	89.1	0.18	1.94	0.2

Total			478		0.18	2	0.2

**Figure 1:**
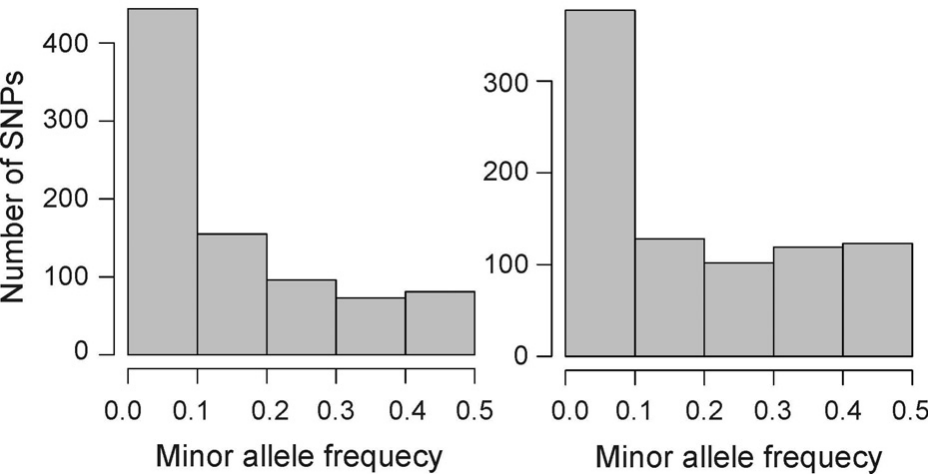
**Distribution of minor allele frequency classes in the populations of spring and winter wheat**.

Significant differences were detected between the distribution of MAF classes in spring and winter wheat (χ^2 ^= 50, *P *= 3.5 × 10^-10^, Figure 1). The spring varieties showed increased proportion of medium frequency alleles with MAF > 0.3.

### Population structure of winter and spring wheat

For the analysis of population structure, the SNP genotyping data was organized into four datasets: three genome-specific datasets for the A- (91 loosely linked SNPs), B- (89 loosely linked SNPs), and D- (39 loosely linked SNPs) genomes and a combined set of 219 SNPs covering the entire wheat genome (see Methods and the Additional File 3.xls). The comparison of admixture models assuming independent or correlated allele frequencies suggest that a model assuming independent allele frequencies is more appropriate than the model with correlated allele frequencies for inferring the number of genetically homogeneous clusters in our dataset. The appropriate choice of the model is strongly influenced by the evolutionary history of populations. The breeding populations include diverse lines subjected to different selection regimes that can result in differentiated allelic frequencies in populations adapted to varying environmental conditions. Falush et al. [43] also pointed out that the correlated allele frequency model may overestimate K when the allele frequencies between populations are different. Since the models assuming independent and correlated allele frequencies produced similar population subdivision and classification of cultivars, hereafter, we describe only results from the independent model unless otherwise noted.

First, we tested whether structure analysis assuming *K *= 2 would assign winter and spring wheat cultivars into two separate clusters. Both frequency models based on the A- and B-genome SNP data produced similar population subdivision. The inferred population structure was consistent across multiple simulation runs. The majority of winter and spring wheat breeding populations were assigned to separate clusters (Additional File 5.xls). Only the A-genome data in the NY winter wheat population showed an equal proportion of ancestry in the two clusters (Additional File 5.xls). Grouping of varieties at *K *= 2 using the D-genome data did not result in clear separation of spring and winter lines. Only 7 out of 17 breeding populations derived more than 80% of their D-genome's genetic ancestry from only one of the two clusters.

To identify the optimal number of *K *clusters in genome-specific datasets, we calculated the posterior probability Pr (K|X) [42] and ΔK [45] for each simulation run. The posterior probability in structure runs was constantly increasing with increasing the values of K ranging from 2 to 21 providing little guidance in selecting the optimal number of clusters. The InStruct software [47] showed a similar trend (data not shown) for the same range of *K *values. These observations were consistent with previously reported analyses of population structure in barley and maize breeding populations using multi-locus SNP data [56,57]. Therefore, the selection of the optimal value of *K *in this study was based on the analysis of relationship between Pr (X|K), value of *K *and the variation of Pr (X|K) among multiple independent runs of Gibb's sampler.

**Figure 2:**
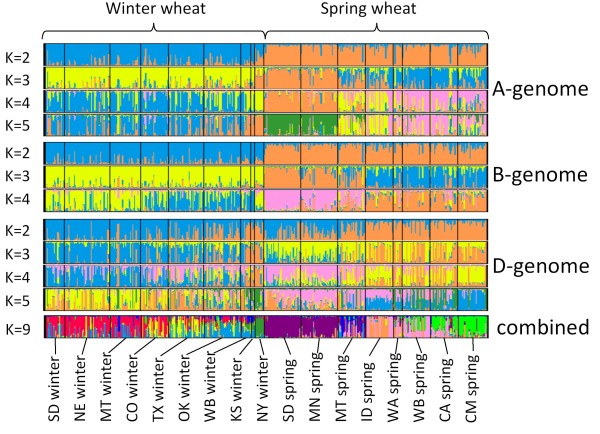
**Population structure of spring and winter wheat lines**. Clustering was performed using A-, B-, D-genome and combined SNP sets. The genotype of each line on the figure is represented by a colored line where each color reflects the membership of a cultivar in one of the K clusters. The proportion of the colored segment indicates the proportion of the genome drawn from the K clusters.

The probability of data for *K *from 2 to 5 for the A-genome SNP dataset was consistent among multiple independent runs of Structure (Figure 2 and Additional File 4.ppt). For the B-genome SNP dataset, we obtained consistent Pr (X|K) for *K *values varying from 2 to 4. For values of *K *above 6 for the A-genome dataset and above 5 for the B-genome dataset the simulation runs could not converge to a single mode (Additional File 4.ppt). The ambiguity of clustering solutions was also accompanied by smaller increase in the mean Pr (X|K). The cluster analysis of both the A- and B-genome datasets showed that the rate of change of Pr (X|K) with increase in *K *reached more or less stationary value (~200) for values of *K *= 4 or higher (Additional File 4.ppt). The maximum likelihoods of clustering obtained for correlated and uncorrelated allele frequency models suggested different values of *K *for the D-genome dataset. The likelihood of the correlated allele frequency model for the D-genome dataset reached its maximum at *K *= 9. However, the rate of likelihood gain decreased for *K *values above 7. The likelihood of independent allele frequency model showed that the improvement of the likelihood of clustering dropped dramatically for *K *above 5.

**Figure 3:**
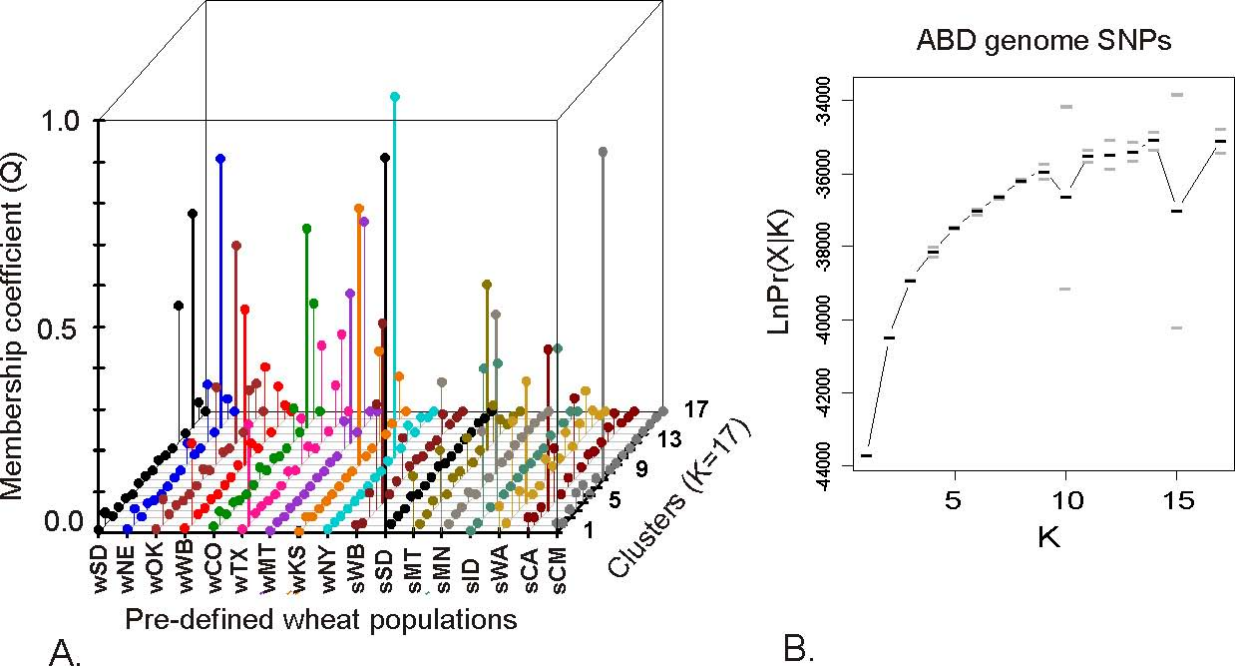
**Clustering of 17 pre-defined wheat populations**. A) Proportion of membership (Q) of 17 pre-defined populations in 17 clusters inferred using the Structure program. Maximum membership coefficients in one of the 17 clusters for each pre-defined population are indicated by thick lines; B) The log probability of data as a function of *K *for genome-wide 219 SNP dataset. Means (black bars) and 95% confidence intervals (grey bars) of log probability of data Ln Pr (X|K) for each value of K were calculated from 10 independent runs of structure with 100,000 burn-in steps and 10^6 ^simulation steps.

The genome-wide set of 219 SNPs was first used for assigning each of the 17 pre-defined populations to separate clusters. It is expected that each population should have maximum membership in only one cluster if the allele frequencies among populations are significantly different. However, the clustering analysis demonstrated that in several cases more than one population had membership in the same cluster (Figure 3A). There were also at least five clusters for which none of the 17 pre-defined populations showed a maximum membership coefficient. The maximum values of population-specific membership coefficients Q suggested that only NY and OK winter wheat populations and SD, CA, CM, MN, and MT spring wheat populations derived the majority of their alleles from a single cluster. These results indicated that 17 clusters exceeded the actual number of genetically distinct populations in our sample. A number of cultivars from the CA spring wheat population share ancestry with the lines from the CIMMYT population and nearly all SD and MN spring cultivars were assigned to the same cluster. The winter wheat populations showed lower levels of genetic differentiation with the majority of cultivars in SD, NE, CO, and MT populations having membership coefficient above 0.5 in the same cluster. A reduction in the number of *K *clusters to ensure that each predefined population has a maximum membership in only one cluster led to the conclusion that optimal clustering can be achieved at *K *= 9 (Figure 2 and Additional File 6.xls). This value of *K *is also supported by the calculation of the likelihood of data (Figure 3B).

The shared ancestry can result from the usage of related cultivars in different breeding programs, which sometimes can be reflected in the pedigree of lines included in our study (Additional File 1.xls). For example, the cultivar Express was used in the spring wheat breeding programs of CA and WestBred, and cultivar Milan was used in the breeding programs of CIMMYT and CA. However, pedigree data clearly shows that only small fraction of cultivars share common parents. Apparently, pedigree has limited power for inferring the level of genetic relatedness among cultivars in breeding programs because: 1) common lines can be used only at the very early stages of cultivar development and derived lines may carry only small fraction of parental genotype; 2) other parents used in a breeding program can have larger contribution to the genotype of cultivar; 3) pedigree has an unknown level of error; 4) some pedigrees may contain incomplete information.

**Figure 4:**
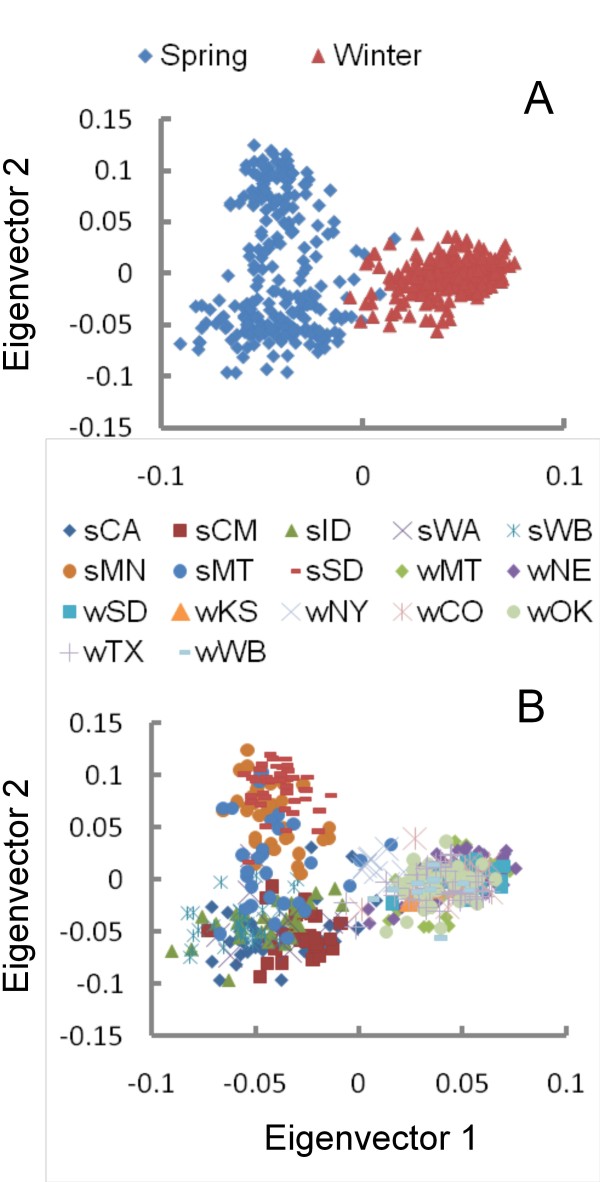
**Principal component analysis of genotyping data**. A) Clustering of winter and spring wheat lines; B) Clustering of pre-defined breeding populations; sCA, sCM, sID, sWA, sWB, sMN, sMT and sSD - spring wheat populations; wMT, wNE, wSD, wKS, wNY, sCO, wOK, wTX and wWB - winter wheat populations.

Principal component analysis (PCA) of population structure revealed that the first two principal components can separate the wheat populations into 3 clusters (Figure 4). The eigenvector 1 separated spring and winter wheat populations; the eigenvector 2 separated spring wheat populations into two clusters, with one cluster predominantly containing lines from MN and SD, while spring lines from CA, CM, ID, WA and WB populations were grouped more closely together. The spring wheat lines from MT, however, showed higher levels of admixture and thus could not be placed within a single cluster (Figure 4).

### Genetic differentiation of populations

Analysis of molecular variance components based on the 849 SNPs showed that wheat lines within breeding populations are more highly genetically differentiated than wheat lines among different populations, which is reflected by a higher proportion of variance within breeding populations than among breeding populations and growth habit groups (Table 3). A higher proportion of among-subpopulation genetic variation was found in spring wheat (17.2%) compared to that in winter wheat (10.6%), indicating that in our panel spring wheat populations are more genetically differentiated than winter wheat populations. Only 9.7% of genetic differentiation among lines was explained by grouping all varieties into spring and winter populations.

**Table 3:** Analysis of molecular variance components.

Sample	No. of growth habits	No. of subpopulations	Variance components (percents of variance)		
			
			Within subpopulations	Among subpopulations within growth habit	Among growth habit
Winter	1	8	89.4%	10.6%	-
Spring	1	8	82.8%	17.2%	-
Combined	2	16	77.4%	12.9%	9.7%

The F_ST _estimates for individual loci between spring and winter wheat populations inferred a high level of heterogeneity across chromosomes (Table 4) with the majority of SNP loci having low F_ST _values (Additional File 7.tif). The substantial variation in F_ST _estimates for single SNP loci is supported by the standard deviations of chromosome- and genome-specific F_ST _estimates exceeding the values of means (Table 4). Similar observation for single-locus F_ST _estimates was previously noted in human populations [58]. We found that the F_ST _estimates for individual genomes were consistent with the results of population structure analysis. A lower level of genetic differentiation between the spring and winter wheat populations was observed in the D-genome (mean F_ST _= 0.07) relative to the A- (mean F_ST _= 0.1) and B-genomes (mean F_ST _= 0.11). Up to 50% reduction in variation of single-locus F_ST _estimates can be achieved by calculating the group means of adjacent SNP markers (Table 4). As shown in the Additional File 7.tif, the mean F_ST _values for windows of 5 SNP loci exhibited a lower proportion of extreme values and a narrower distribution. However, the fact that significant variation was still observed for window-based F_ST _estimates suggests that similar F_ST _values are clustered according to their genomic location.

**Table 4:** Means and standard deviations of single-locus FST estimates obtained for spring and winter wheat populations.

Chromosome	**Mean F**_**ST**_	Standard deviation (single-locus estimates/5 SNP windows)	Genome	**Mean F**_**ST**_	Standard deviation (single-locus estimates/5 SNP windows)
1A	0.06	(0.08/0.04)			
2A	0.11	(0.15/0.08)			
3A	0.08	(0.1/0.04)			
4A	0.11	(0.14/0.08)			
5A	0.21	(0.27/0.16)			
6A	0.05	(0.07/0.03)			
7A	0.06	(0.07/0.05)	A	0.10	(0.15/0.09)

1B	0.07	(0.07/0.03)			
2B	0.14	(0.16/0.1)			
3B	0.14	(0.14/0.05)			
4B	0.03	(0.03/0.02)			
5B	0.08	(0.08/0.04)			
6B	0.12	(0.13/0.1)			
7B	0.14	(0.16/0.09)	B	0.11	(0.13/0.08)

1D	0.10	(0.11/0.04)			
2D	0.06	(0.07/0.03)			
3D	0.00	(0.01/NA)			
4D	0.08	(0.11/NA)			
5D	0.02	(0.01/)			
6D	0.10	(0.13/0.03)			
7D	0.03	(0.06/0.05)	D	0.07	(0.1/0.05)

Means of F_ST _estimates in windows of 5 loci were used to identify the regions of the wheat genome which are genetically differentiated between spring and winter wheat populations. Although this approach cannot be considered a formal test for selection, it can be used as a preliminary test to identify genomic regions harboring genes controlling plant growth habit. Random permutation of genome-wide single-locus F_ST _values was used to estimate 95% confidence interval (CI) for window-based F_ST _values. The confidence interval was used as a threshold to identify regions showing F_ST _values higher than the genome-wide mean. Seven regions with F_ST _values higher than 95% CI were detected on chromosomes 2A, 2B, 5A, 6B and 7B (Table 5). Comparison of these regions with previous genetic studies of flowering in wheat showed that three out of the seven regions overlap with previously mapped flowering time QTL or cloned flowering genes [59-61].

**Table 5:** Genetic map intervals that show elevated levels of FST and overlap with flowering time QTL.

	Chromosome arm	Interval, cM	**Mean F**_**ST**_	Known genes involved in growth habit phenotype
Region 1	2AL	114-124	0.27	
Region 2	2BS	76-97	0.24	Ppd-B1 [59]
Region 3	2BL	104-110	0.32	
Region 4	5AL	35-46	0.30	
Region 5	5AL	80-138	0.44	Vrn-A1 [60]
Region 6	6BS/6BL	72-75	0.28	
Region 7	7BL	63-89	0.28	Flowering time QTL [61]

### Patterns of linkage disequilibrium across the wheat genome

A total of 394 genetically mapped SNPs were used for estimating the extent of LD in wheat populations. Only SNP loci having MAF ≥ 0.05 in a particular population were used for analysis. Loci mapped to the regions of the wheat genome known to be subjected to structural alterations during evolution were excluded from this analysis [62]. In the spring and winter wheat populations, the average extent of significant intra-chromosomal LD (FDR ≤ 0.01) was 20.8 cM (median 11.5 cM) and 19.2 cM (median 10.7 cM), respectively. Significant LD was observed for 68% (253/370) of SNP loci in spring wheat and for 71% (247/348) of SNP loci in the winter wheat. Significant LD was detected for 8.9% and 5.7% of unlinked SNP loci (located on different chromosomes) in the spring and winter wheat populations, respectively. In the combined population of 478 lines, the mean intra-chromosomal LD extended over 19.5 cM (median 10.7 cM) and significant associations were discovered in 8.1% of unlinked loci.

The highest extent of significant LD in the spring, winter and combined populations was observed in the wheat D-genome (mean 20.8 - 27.1 cM), followed by lower LD in the A- (mean 16.7 - 19.8 cM) and B-genomes (mean 20.4 - 21.5 cM) (Additional File 8.xls). The estimates of median LD for significant associations were similar among the wheat genomes and populations, suggesting that the differences among estimates of mean LD were driven by population- and genome-specific differences in the long-range LD, which in most cases exceed 10 cM.

**Figure 5:**
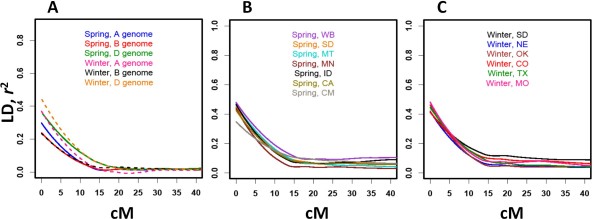
**LD decay estimated using the wheat A-, B-, and D-genome and combined SNP sets**. A) Decay of *r*^2 ^as a function of genetic distance between SNP markers estimated for A-, B-, and D-genomes of the spring and winter wheat populations. B) Decay of *r*^2 ^as a function of genetic distance between SNP markers estimated for the spring wheat populations from different geographic location in US and Mexico. B) Decay of *r*^2 ^as a function of genetic distance between SNP markers estimated for the winter wheat populations from different geographic location in US. Only populations with more than 20 wheat lines were included in these analyses.

The estimates of *r*^2 ^for all pairs of linked 394 SNP loci were used to assess the rate of LD decay with genetic distance. The statistically significant threshold for *r*^2 ^in the spring and winter wheat populations was 0.08 and 0.02, respectively. In the A-genome, LD declined to 50% of its initial value at about 5 cM in winter wheat compared to 6.3 cM in spring wheat (Figure 5A). In the D-genome, LD decayed faster in winter wheat declining to 50% of its initial value at about 6 cM whereas in spring wheat a similar level of LD was reached at 7 cM (Figure 5A). Both spring and winter wheat populations showed an identical rate of LD change in the B-genome decaying to 50% of its initial value over 7 cM (Figure 5A).

In order to investigate population-specific recombination processes we studied the rate of LD decay within the populations of different origin (Figure 5B and 5C). Due to the limited number of lines, WB, KS and NY winter wheat and WA spring wheat populations were excluded from this analysis. As expected, compared to LD estimates in the combined population dataset (Figure 5A), the estimates of LD within populations were higher (Figure 5B and 5C). The level of initial LD (the highest value of LD on Figure 5) in spring wheat populations, except for the CIMMYT population, varied from 0.43 to 0.49 (Figure 5B). The CIMMYT population had the lowest level of initial LD (*r*^2 ^= 0.35) decaying to 50% of its value at about 10.5 cM. In the remaining spring wheat populations LD decayed to half of its initial value within 6-9 cM range (Figure 5B). The levels of initial LD in the winter wheat populations were similar to those in the spring wheat population with *r*^2 ^ranging from 0.42 to 0.49 (Figure 5C) and decaying to 50% of these initial values within 7-9 cM.

**Figure 6:**
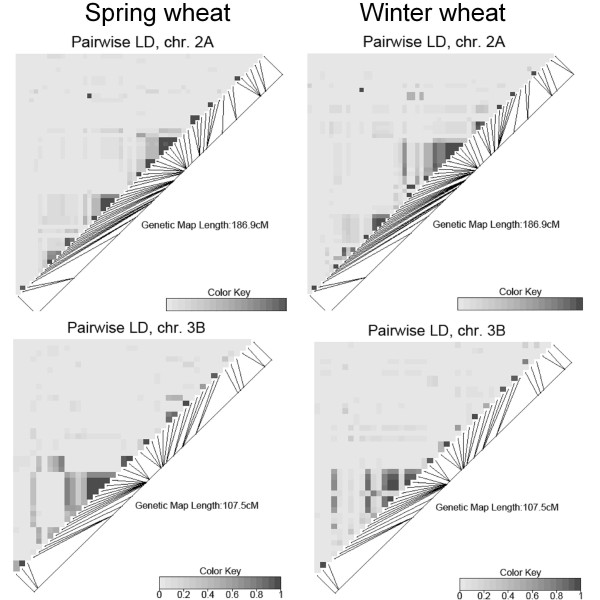
**Pair-wise LD for the wheat chromosomes 2A and 3B**. Colored rectangles represent the squared correlation *r*^2 ^between a pair of SNPs. The values of *r*^2 ^are color-coded according to the color-key provided below the LD maps.

The extent of LD varied greatly across the wheat genome even among closely linked SNP loci. Using the method described by Gabriel et al. [54], we identified only two genomic regions harboring 5 or more SNP loci that show little evidence of historical recombination events. These LD blocks shared by winter and spring wheat populations were located on chromosomes 2A and 3B (Figure 6). Pairs of loci with elevated LD tended to be localized near the centromere in regions bearing low recombination rates. The LD data for each chromosome in the population of 478 wheat cultivars are summarized in the Additional File 9.xls.

## Discussion

### Genetic diversity and population structure

Our study provides an overview of genetic variation in US and CIMMYT spring and winter wheat cultivars using genome-wide distributed SNP markers. Here we confirmed the utility of the wheat OPA for genotyping large populations of hexaploid wheat lines [32]. Most of the SNPs that were polymorphic within the complete set of 478 cultivars were also polymorphic in all subpopulations. Of the 849 polymorphic SNPs, 89% were polymorphic in both spring and winter wheat populations and from 70% to 85% were polymorphic across populations. Such a widespread distribution of polymorphic loci among populations suggests that the SNP discovery performed in a set of genetically diverse wheat landraces and wild emmer wheat [19,36] was successful in recovering alleles represented in both growth habit groups. However, the distribution of MAF showed a higher proportion of medium frequency alleles in the spring wheat than in the winter wheat population. Currently, it is not clear whether this observed bias is caused by historical events, such as demography or selection, or if it is the result of ascertainment schemes applied during SNP discovery process. If the latter is true, the bias is likely small given the high proportion of polymorphic SNPs shared between spring and winter populations.

The proportion of genetic differentiation explained by growth habit (9.7%) was only slightly lower than the proportion of variation among subpopulations within the growth habit groups (12.9%). Historical gene flow between the spring and winter wheat groups during crop improvement and breeding can potentially be responsible for the low level of genetic differentiation between these populations. The proportion of variance between growth habit and among populations was significantly lower than the within-population genetic variance, indicating that each of the breeding programs included in our study employs genetically diverse lines. These results also indicate that the polymorphic SNPs included in the wheat OPA are represented in most populations and, therefore, will be useful for genotyping diverse collections of wheat cultivars.

In spite of the high proportion of shared SNPs among populations as well as small among-population genetic variance components, the model-based clustering approach was able to successfully assign cultivars to clusters. The clustering analysis performed using the whole genome SNP set produced more genetically distinct clusters than clustering obtained with smaller sets of SNPs from the A-, B- or D- genomes. Although cultivars can be optimally clustered at the same value of *K *using the A- and D- genome SNP sets, the proportion of genetic ancestry of cultivars in these clusters was variable for different SNP sets, implying that the three wheat genomes have different degrees of genetic differentiation among breeding programs. This outcome may be a consequence of inadequate representation of SNP alleles within a particular genome in different populations, or alternatively, can reflect the different impact that demography, population and breeding history had on genomes of wheat lines. Strong selection for adaptation to diverse environmental conditions, together with different founders and introgression histories can modulate the differentiation of allelic frequencies among breeding populations and genomes and result in the slightly different clustering patterns obtained here using the three genome-specific SNP sets. A similar trend was documented for US, Australian and UK varieties using DArT markers [63], which showed that wheat genomes are differentiated in allelic frequency among national breeding programs [64].

Even when the full SNP data set was used, wheat cultivars in the 17 wheat populations rarely shared the same membership coefficient in the inferred clusters reflecting the complexity of the breeding histories of lines included in this analysis. Most wheat lines showed evidence of admixture with the portions of their genomes assigned to 2 - 4 different inferred clusters, which is an expected result of the frequent crosses used in wheat breeding programs between adapted germplasm and the donors of different traits. When the whole population was forced to divide into two groups (K = 2), the clusters aligned mainly by growth habit, with most spring and winter wheat cultivars being assigned to separate clusters. The same grouping by growth habit was also apparent when the analyses were performed separately for the A- and B-genome SNPs. However, the informativeness of D-genome SNPs for the separation of spring and winter varieties was low, likely a consequence of low allelic frequency differentiation between these two wheat groups as evidenced by the low inter-population F_ST _obtained for the D-genome.

Clustering using the combined SNP set showed that the inferred number of clusters in our population is smaller than the number of pre-defined breeding populations, largely due to the fact that breeding programs from the same region tend to use cultivars of common ancestry. The spring wheat population included cultivars from breeding programs targeting more geographically separated areas that were also more genetically differentiated that the winter wheat population. A high level of genetic differentiation was observed between the populations originating from two major geographical locations one including northern states SD, MN and MT and the other including Mexico and western states WA, ID and CA. In contrary, the winter wheat populations largely originating from the central states showed higher levels of admixture and a lower extent of genetic differentiation.

### Genetic differentiation of spring and winter wheat

The characterization of F_ST _across chromosomes provided additional insights into the structure of genetic variation between the spring and winter wheat populations. Assuming the same evolutionary processes affect neutral loci, identifying genomic regions showing elevated F_ST _between spring and winter wheat populations should make it possible to localize the targets of selection controlling growth habit phenotype. However, the substantial heterogeneity of F_ST _estimates for SNP loci across the wheat genome make it impossible to use single-locus F_ST _values for detecting past selection events. This problem was circumvented by calculating F_ST _for a group of sequential SNP loci which was shown to be an efficient strategy to reduce variation in F_ST _estimates relative to estimates based on individual loci [58]. The highest degree of genetic differentiation was identified for the loci mapped to the wheat chromosome 5A, which probably results from the presence of *Vrn1 *gene locus, the major gene involved in regulation of flowering time in wheat [60]. This locus is responsible for most of the natural variation in the growth habit in hexaploid wheat [65,66]. Additional regions showing unusually high level of genetic differentiation between spring and winter wheat lines were detected on the chromosomes 2A, 2B, 6B and 7B (Table 5). Three out of seven regions with elevated F_ST _were co-localized with previously mapped genes known to be involved in flowering time regulation. Some wheat chromosome 6B substitution lines are known to affect flowering time in the absence of vernalization [67], but since the responsible gene has not been mapped, it is not possible to determine if this gene locus overlaps with high F_ST _region identified on the chromosome 6B in this study. Although the distribution of empirical F_ST _estimates cannot serve as a formal test for selection, this finding suggests that high F_ST _genomic regions can harbor genes subject to diversifying selection providing good targets for further studies.

The genetic differentiation of some of the genomic regions can also be due to structural rearrangements abundant in one of the populations. For example, chromosomal inversions are known to be a major barrier for gene flow between populations due to limited recombination near the affected genomic regions and also one of the mechanisms facilitating reproductive isolation and species formation [68]. Previously it was demonstrated that pericentomeric inversion polymorphisms are widespread in wheat [69]. We found that one of these inversions overlaps with one of the regions with elevated F_ST _detected on the chromosome 6B. This structural rearrangement can potentially impact the frequency of allele exchange between the spring and winter wheat populations and contribute to the genetic differentiation of this genomic region.

### Linkage disequilibrium

Using genome-wide SNP data we demonstrated the extensive amount of LD in the populations of wheat cultivars. The variation in the patterns of LD among the populations and wheat genomes reflects the complexity of evolutionary and breeding history of wheat [70]. The extent of LD and LD decay estimated using SNP loci combined from all three wheat genomes was similar in both spring and winter wheat populations. In the analyses using the individual genomes, the differences between spring and winter lines in LD decay to 50% were also very small varying from no difference in the B-genome (7 cM both) to 1.3 cM in the A-genome (6.3 cM spring and 5 cM winter).

Analyses of LD decay by breeding population showed similar profiles among populations except for the CIMMYT population, which had the lowest LD among completely linked loci and the slowest rate of LD decay. A possible explanation for this observation is the intensive usage of synthetic wheat lines in the CIMMYT program. Synthetic wheats are generated by hybridization of diverse tetraploid (A- and B-genomes) and *Ae. tauschii *(D-genome) accessions followed by chromosome duplication using colchicine. The synthetic wheats and their derivatives have greatly increased genetic diversity in hexaploid wheat, particularly in the D-genome [71-73]. It is well known that the introduction of new haplotypes from divergent populations can increase the extent of LD [74].

Depending on the genomic location of genes controlling important adaptive traits, these broad crosses can have a differential impact on LD in different genomes. For example, because the *Vrn-A1 *gene has a stronger effect than the *Vrn-B1 *gene, it has higher number of widely distributed haplotypes [66] and is thus more likely to have a stronger effect on LD. Therefore, the divergence in the extent of LD between wheat populations is probably related to unique breeding histories and selection pressures applied to genes located in the different genomes during the process of cultivar development.

A genetic bottleneck may also increase the level of LD [2,74]. The last polyploidization event resulting in the origin of hexaploid bread wheat approximately 8,000-10,000 years ago had a dramatic impact on the level of genetic diversity in the D-genome [19,75] suggestive of strong population bottleneck. We hypothesize that the longer extent of significant LD in the D-genome compared to that in the A- and B-genomes in both spring and winter wheat populations can mostly be explained by this polyploidization event [19]. However, the difference in LD between the D-genome, and the A- and B-genomes in spring wheat was not as high as in winter wheat. This result can probably be explained by 1) the larger number of breeding cycles involved in the development of spring wheat cultivars than in the development of winter wheat cultivars, and/or by 2) the inclusion of synthetic-derived wheat cultivars in the CIMMYT spring population.

Rates of LD decay varied among populations, but as expected, individual populations showed higher overall levels of LD than the combined datasets. These higher LD levels were also reflected in elevated levels of long-range LD extending above 10 cM. Interestingly, across all populations, LD decayed to 50% of its initial value within relatively narrow genetic intervals ranging from 6 to 9 cM. This rate of LD decay is probably associated with the high level of genetic diversity used in the individual breeding program. The cultivars in all of these programs captured comparable number of recombination events resulting in fast erosion of LD. However, each population showed variation in the extent of long range LD which was highest in the SD winter wheat population and WB spring wheat population. As pointed out earlier, these differences are probably the consequence of breeding history and selection specific to each breeding program.

The comparison of the LD levels obtained in our study with results obtained in other studies dealing with wheat and other inbreeding crops was complicated by the differences in the type of markers used for genotyping, and by sample size variation in the different studies. Both factors can impact LD estimates. Previously reported LD estimates in wheat were obtained using more polymorphic SSR markers [22,24,25]. In a sample of 43 U.S. spring and winter wheat cultivars it was shown that 70 out of 123 SSR loci (57%) with significant LD were linked at <10 cM [22]. In our study 86% of SNP loci (211/246) showing significant LD in the combined population of spring and winter wheat were located at less than 10 cM. The larger proportion of alleles with significant LD at less than 10 cM detected in our study is most likely due to sample size differences across the two studies (478 vs. 43 lines) used to estimate significant LD. The extent of significant LD in our population was more than 4 times higher than the SNP-based estimates obtained for a population of 91 European spring and winter cultivated barley [3]. These results suggest that the genetic diversity and number of recombination events in European barley germplasm are significantly higher than in the sample of U.S. and CIMMYT wheat cultivars. Therefore, association mapping studies in wheat would require a smaller number of markers per unit of genetic distance than needed in cultivated barley.

Variation in the extent of LD along the chromosome affect the number of tagSNPs (subset of SNPs that capture a large fraction of the allelic variation of all SNP loci [76]) required in each genomic region to ensure that causal mutations are in LD with neighboring SNPs. The interaction of many factors affecting the rate of LD decay in the different parts of the genome complicates the determination of the number of tagSNPs required to gain sufficient power for genome-wide association mapping. Estimates of this number for autogamous plant species varied from 9,600 to 29,400 SNPs for soybean cultivars [6] to 250,000 for the more diverse Arabidopsis natural populations [4]. LD values of 0.8 or higher have been recommended as an acceptable threshold for tagSNP selection [77]. In our study, for loci located from 0.0 to 0.2 cM apart, the median LD was approximately 0.8. If markers are evenly distributed at 0.2 cM intervals, the causative mutation would be found at about 0.1 cM from one of the flanking markers and have an approximate LD of 0.8. In a 3,500 cM hexaploid wheat map, placing markers at 0.2 cM will require at least 17,500 markers. This number would vary depending on if more liberal or conservative LD thresholds were selected.

The evolutionary history of an allele also has a strong impact on the probability of detecting marker-trait associations. Alleles of loci that are involved in local adaptation and subjected to recent selection can be more readily detected using an even more sparsely distributed set of markers. For example, marker-trait associations of alleles involved in the regulation of flowering time in Arabidopsis [78] and cultivated barley [3] were detected using a relatively low number of SNP markers. Genome-wide re-sequencing efforts similar to the ones performed for Arabidopsis [4], rice [1] and maize [79] will be required to provide comprehensive information for tagSNP selection in wheat. These efforts will also need to be complemented by assessment of the portability of selected tagSNPs to other populations. Otherwise, inadequate genome coverage may result in failure to identify critical associations [78,80]. The possibility of performing GWAM in the large polyploid wheat genome will be tested in future using a larger panel of up to 9,000 genome-wide distributed SNP markers currently under development.

## Conclusions

Our study demonstrated a high level of genetic diversity and relatively fast decay of LD within each wheat breeding program. Extensive exchange of genetic material between breeding programs resulted in a low level of genetic differentiation between populations of spring and winter wheat. The regions of the wheat genome harboring flowering time QTL demonstrated the highest levels of genetic differentiation between the spring and winter wheat populations. Breeding, selection and founder effect had a different impact on the wheat genomes in distinct populations, highlighting the significance of allopolyploidy for the development of cultivars adapted to a broad range of environmental conditions. Assessment of the extent of LD and population structure in the assembled panel provided valuable information for the design of GWAM experiments in wheat.

## Authors' contributions

EDA and JDu designed research; EDA coordinated research activity; EDA performed bioinformatical analysis of data and the design of wheat OPA; JDv, MCL, and EDA generated the genetic map; SC performed genotyping; EDA and SC analyzed genotyping data; JDu, SPB, RM, DRC, LET, JAA, SD, KG, JC, KC, PLB, JCR, SH, BFC, SP and MES performed selection and assembly of the panels of spring and winter wheat lines; EDA drafted the manuscript; JDu, JDv, SC, JC, JAA, MES, SH, SPB, SD, LET worked on the preparation of the final version of the manuscript; all authors read and approved the final version of the manuscript.

## Supplementary Material

Additional file 1 **Complete list of wheat cultivars used in the study**. The file contains the list of spring and winter wheat cultivars selected from 17 breeding programs in US and CIMMYT. Pedigree (when available/known) of each cultivar is also provided. Click here for file

Additional file 2 **SNPs and their flanking sequences used for the design of wheat OPA**. The file contains the list of SNPs and their flanking sequences used for the design of wheat Illumina OPA. The Illumina^® ^Assay Design Tool was used to generate designability rank scores for each SNP. Click here for file

Additional file 3 **List of 219 SNPs used for population structure analysis**. The file contains the list of 219 SNPs and their genetic map locations. The analysis of population structure was performed using all SNPs and SNPs separated into genome-specific sets (91 A-genome specific SNPs, 89 B-genome specific SNPs, and 39 D-genome specific SNPs). Click here for file

Additional file 4 **Relationship between the log probability of data and the number of clusters K**. The log probability of data (Ln Pr(X|K)) was plotted as a function of the number of clusters K for different SNP datasets and structure models assuming correlated (top three graphs) and independent (bottom three graphs) alleles frequencies. Means (black bars) and 95% confidence intervals (grey bars) of log probability of data Ln Pr(X|K) for each value of K were calculated from 10 independent runs of Structure with 100,000 burn-in steps and 10^6 ^simulation steps. Click here for file

Additional file 5 **Membership coefficients of 17 pre-defined wheat populations in 2 clusters (K = 2)**. Clustering was estimated using SNPs mapped to the A-, B- and D-genomes. Membership coefficients were calculated from 10 independent runs of Structure with 100,000 burn-in steps and 10^6 ^simulation steps. Click here for file

Additional file 6 **Membership coefficients of 17 pre-defined wheat populations in 9 clusters (K = 9)**. Membership coefficients (Q) were estimated for 17 wheat populations assuming 9 clusters in data (*K *= 9). Clustering was estimated using combined set of 219 SNPs from 10 independent runs of Structure with 100,000 burn-in steps and 10^6 ^simulation steps. Click here for file

Additional file 7 **Distribution of F_ST _estimates for individual SNP loci and windows of 5 SNPs**. **A) **The distribution of single-locus F_ST _values between spring and winter wheat populations. **B) **The distribution of F_ST _values in a sliding window of 5 consecutively located SNP loci. Click here for file

Additional file 8 **Summary of significant LD in the spring, winter and combined populations**. The file contains mean and median estimates of statistically significant LD in the A- B- and D-genomes of spring, winter and combined populations. The pair-wise LD was measured using the squared allele-frequency correlations *r*^2 ^according to Weir [50]. The statistical significance of individual *r*^2 ^estimates was calculated by the exact test following the procedure described by Weir [50]. The false discovery rate (FDR) was established at 0.01 using the Benjamini & Hochberg method [52]. Click here for file

Additional file 9 **Summary of LD estimates**. The file contains the exact test for LD, genetic distances between pairs of SNP markers and minor allele frequencies (MAF) of alleles used for LD calculation. The pair-wise LD was measured using the squared allele-frequency correlations *r*^2 ^according to Weir [50]. The statistical significance of individual *r*^2 ^estimates was calculated by the exact test following the procedure described by Weir [50]. The false discovery rate (FDR) was established at 0.01 using the Benjamini & Hochberg method [52]. Click here for file

## References

[B1] McNally KL Childs KL Bohnert R Davidson RM Zhao K Ulat VJ Zeller G Clark RM Hoen DR Bureau TE Stokowski R Ballinger DG Frazer KA Cox DR Padhukasahasram B Bustamante CD Weigel D Mackill DJ Bruskiewich RM Rätsch G C Robin Buell CR Leung H Leach JE Genome wide SNP variation reveals relationships among landraces and modern varieties of rice Proc Natl Acad Sci USA 2009 106 12273 12278 10.1073/pnas.0900992106 19597147 PMC2718348

[B2] Wright SI Bi IV Schroeder SG Yamasaki M Doebley JF McMullen MD Gaut BS The effects of artificial selection on the maize genome Science 2005 308 1310 1314 10.1126/science.1107891 15919994

[B3] Rostoks N Ramsay L Mackenzie K Cardle L Bhat PR Roose ML Svensson JT Stein N Varshney RK Marshall DF Graner A Close TJ Waugh R Recent history of artificial outcrossing facilitates whole-genome association mapping in elite inbred crop varieties Proc Natl Acad Sci USA 2006 103 18656 18661 10.1073/pnas.0606133103 17085595 PMC1693718

[B4] Kim S Plagnol V Hu TT Toomajian C Clark RM Ossowski S Ecker JR Weigel D Nordborg M Recombination and linkage disequilibrium in Arabidopsis thaliana Nat Genet 2007 39 1151 1155 10.1038/ng2115 17676040

[B5] Buckler ES 4 Thornsberry JM Plant molecular diversity and applications to genomics Curr Opin Plant Biol 2002 5 107 111 10.1016/S1369-5266(02)00238-8 11856604

[B6] Hyten DL Choi IY Song Q Shoemaker RC Nelson RL Costa JM Specht JE Cregan PB Highly variable patterns of linkage disequilibrium in multiple soybean populations Genetics 2007 175 1937 1944 10.1534/genetics.106.069740 17287533 PMC1855121

[B7] Palaisa K Morgante M Tingey S Rafalski A Long-range patterns of diversity and linkage disequilibrium surrounding the maize Y1 gene are indicative of an asymmetric selective sweep Proc Natl Acad Sci USA 2004 101 9885 9890 10.1073/pnas.0307839101 15161968 PMC470768

[B8] Kane NC Rieseberg LH Selective sweeps reveal candidate genes for adaptation to drought and salt tolerance in common sunflower, Helianthus annuus Genetics 2007 175 1823 1834 10.1534/genetics.106.067728 17237516 PMC1855101

[B9] Caicedo AL Williamson SH Hernandez RD Boyko A Fledel-Alon A York TL Polato NR Olsen KM Nielsen R McCouch SR Bustamante CD Purugganan MD Genome-wide patterns of nucleotide polymorphism in domesticated rice PLoS Genet 2007 3 1745 1756 10.1371/journal.pgen.0030163 17907810 PMC1994709

[B10] Hyten DL Song Q Zhu Y Choi IY Nelson RL Costa JM Specht JE Shoemaker RC Cregan PB Impacts of genetic bottlenecks on soybean genome diversity Proc Natl Acad Sci USA 2006 103 16666 16671 10.1073/pnas.0604379103 17068128 PMC1624862

[B11] Luo MC You FM Kawahara T Waines JG Dvorak J The structure of wild and domesticated emmer wheat populations, gene flow between them, and the site of emmer domestication Theor Appl Genet 2007 114 947 959 10.1007/s00122-006-0474-0 17318496

[B12] Stracke S Presterl T Stein N Perovic D Ordon F Graner A Effects of introgression and recombination on haplotype structure and linkage disequilibrium surrounding a locus encoding Bymovirus resistance in barley Genetics 2007 175 805 817 10.1534/genetics.106.063800 17151251 PMC1800611

[B13] Lexer C Buerkle CA Joseph JA Heinze B Fay MF Admixture in European Populus hybrid zones makes feasible the mapping of loci that contribute to reproductive isolation and trait differences Heredity 2007 98 74 84 10.1038/sj.hdy.6800898 16985509

[B14] Randi E Detecting hybridization between wild species and their domesticated relatives Mol Ecol 2008 17 285 293 10.1111/j.1365-294X.2007.03417.x 18173502

[B15] Haudry A Cenci A Ravel C Bataillon T Brunel D Poncet C Hochu I Poirier S Santoni S Glémin S David J Grinding up wheat: a massive loss of nucleotide diversity since domestication Mol Biol Evol 2007 24 1506 1517 10.1093/molbev/msm077 17443011

[B16] Buckler ES Thornsberry JM Kresovich S Molecular diversity, structure and domestication of grasses Genet Res 2001 77 213 218 10.1017/S0016672301005158 11486504

[B17] Willcox G Damania AB, Valkoun J, Willcox G, Qualset CO Archaeobotanical evidence for the beginnings of agriculture in Southwest Asia The Origins of Agriculture and Crop Domestication 1997 ICARDA, IPGRI, FAO and UC/GRCP, ICARDA, Aleppo, Syria 25 38

[B18] Dvorak J Luo MC Akhunov ED N.I. Vavilov's theory of centers of diversity in the light of current understanding of wheat domestication and evolution Proceedings of the 8th International Wheat conference 2010 Saint-Petersburg

[B19] Akhunov ED Akhunova AR Anderson OD Anderson JA Blake N Clegg MT Coleman-Derr D Conley EJ Crossman CC Deal KR Dubcovsky J Gill BS Gu YQ Hadam J Heo H Huo N Lazo GR Luo MC Ma YQ Matthews DE McGuire PE Morrell PL Qualset CO Renfro J Tabanao D Talbert LE Tian C Toleno DM Warburton ML You FM Wheat nucleotide diversity maps reveal variation in diversity among wheat genomes and chromosomes BMC Genomics 2010 11 702 21156062 10.1186/1471-2164-11-702 PMC3022916

[B20] Kihara H Discovery of the DD-analyser, one of the ancestors of Triticum vulgare (Japanese) Agric and Hort (Tokyo) 1944 19 13 14

[B21] McFadden ES Sears ER The origin of Triticum spelta and its free-threshing hexaploid relatives J Hered 1946 37 81 89 20985728 10.1093/oxfordjournals.jhered.a105590

[B22] Chao S Zhang W Dubcovsky J Sorrells M Evaluation of genetic diversity and genome-wide linkage disequilibrium among U.S. wheat (*Triticum aestivum *L.) germplasm representing different market classes Crop Sci 2007 47 1018 1030 10.2135/cropsci2006.06.0434

[B23] Raquin AL Brabant P Rhoné B Balfourier F Leroy P Goldringer I Soft selective sweep near a gene that increases plant height in wheat Mol Ecol 2008 17 741 756 10.1111/j.1365-294X.2007.03620.x 18194170

[B24] Maccaferri M Sanguineti MC Noli E Tuberosa R Population structure and long-range linkage disequilibrium in a durum wheat elite collection Mol Breeding 2005 15 271 290 10.1007/s11032-004-7012-z

[B25] Breseghello F Sorrells ME Association mapping of kernel size and milling quality in wheat (Triticum aestivum L.) cultivars Genetics 2006 172 1165 1177 10.1534/genetics.105.044586 16079235 PMC1456215

[B26] Zhao K Aranzana MJ Kim S Lister C Shindo C Tang C Toomajian C Zheng H Dean C Marjoram P Nordborg M An Arabidopsis example of association mapping in structured samples PLoS Genet 2007 3 e4 10.1371/journal.pgen.0030004 17238287 PMC1779303

[B27] Yu J Buckler ES Genetic association mapping and genome organization of maize Curr Opin Biotechnol 2006 17 155 160 16504497 10.1016/j.copbio.2006.02.003

[B28] Crossa J Burgueño J Dreisigacker S Vargas M Herrera-Foessel SA Lillemo M Singh RP Trethowan R Warburton M Franco J Reynolds M Crouch JH Ortiz R Association analysis of historical bread wheat germplasm using additive genetic covariance of relatives and population structure Genetics 2007 177 1889 1913 10.1534/genetics.107.078659 17947425 PMC2147943

[B29] McMullen MD Kresovich S Villeda HS Bradbury P Li H Sun Q Flint-Garcia S Thornsberry J Acharya C Bottoms C Brown P Browne C Eller M Guill K Harjes C Kroon D Lepak N Mitchell SE Peterson B Pressoir G Romero S Oropeza Rosas M Salvo S Yates H Hanson M Jones E Smith S Glaubitz JC Goodman M Ware D Genetic properties of the maize nested association mapping population Science 2009 325 737 740 10.1126/science.1174320 19661427

[B30] Buckler ES Holland JB Bradbury PJ Acharya CB Brown PJ Browne C Ersoz E Flint-Garcia S Garcia A Glaubitz JC Goodman MM Harjes C Guill K Kroon DE Larsson S Lepak NK Li H Mitchell SE Pressoir G Peiffer JA Rosas MO Rocheford TR Romay MC Romero S Salvo S Sanchez Villeda H da Silva HS Sun Q Tian F Upadyayula N The genetic architecture of maize flowering time Science 2009 325 714 718 10.1126/science.1174276 19661422

[B31] Kruglyak L The road to genome-wide association studies Nat Rev Genet 2008 9 314 318 10.1038/nrg2316 18283274

[B32] Akhunov ED Nicolet C Dvorak J Single nucleotide polymorphism genotyping in polyploid wheat with Illumina GoldenGate assay Theor Appl Genet 2009 119 507 517 10.1007/s00122-009-1059-5 19449174 PMC2715469

[B33] Close TJ Bhat PR Lonardi S Wu Y Rostoks N Ramsay L Druka A Stein N Svensson JT Wanamaker S Bozdag S Roose ML Moscou MJ Chao S Varshney RK Szucs P Sato K Hayes PM Matthews DE Kleinhofs A Muehlbauer GJ DeYoung J Marshall DF Madishetty K Fenton RD Condamine P Graner A Waugh R Development and implementation of high-throughput SNP genotyping in barley BMC Genomics 2009 10 582 10.1186/1471-2164-10-582 19961604 PMC2797026

[B34] Hyten DL Song Q Choi IY Yoon MS Cregan PB High-throughput genotyping with the GoldenGate assay in the complex genome of soybean Theor Appl Genet 2008 116 945 952 10.1007/s00122-008-0726-2 18278477

[B35] Dvorak J McGuire PE Cassidy B Apparent sources of the A genomes of wheats inferred from the polymorphism in abundance and restriction fragment length of repeated nucleotide sequences Genome 1988 30 680 689

[B36] Wheat SNP database http://probes.pw.usda.gov:8080/snpworld/Search

[B37] TREP database http://wheat.pw.usda.gov/ITMI/Repeats/index.shtml

[B38] GIRI database http://www.girinst.org/

[B39] Shen R Fan JB Campbell D Chang W Chen J Doucet D Yeakley J Bibikova M Wickham Garcia E McBride C Steemers F Garcia F Kermani BG Gunderson K Oliphant A High-throughput SNP genotyping on universal bead arrays Mutat Res 2005 573 70 82 15829238 10.1016/j.mrfmmm.2004.07.022

[B40] Anderson JA Churchill GA Autrique JE Tanksley SD Sorrells ME Optimising parental selection for genetic linkage maps Genome 1993 36 181 186 10.1139/g93-024 18469981

[B41] Liu K Muse SV PowerMarker: an integrated analysis environment for genetic marker analysis Bioinformatics 2005 21 2128 2129 10.1093/bioinformatics/bti282 15705655

[B42] Pritchard JK Stephens M Donnelly P Inference of population structure using multilocus genotype data Genetics 2000 155 945 59 10835412 10.1093/genetics/155.2.945 PMC1461096

[B43] Falush D Stephens M Pritchard JK Inference of population structure: Extensions to linked loci and correlated allele frequencies Genetics 2003 164 1567 1587 12930761 10.1093/genetics/164.4.1567 PMC1462648

[B44] Jakobsson M Rosenberg NA CLUMPP: a cluster matching and permutation program for dealing with label switching and multimodality in analysis of population structure Bioinformatics 2007 23 1801 1806 10.1093/bioinformatics/btm233 17485429

[B45] Evanno G Regnaut S Goudet J Detecting the number of clusters of individuals using the software STRUCTURE: a simulation study Mol Ecol 2005 14 2611 2620 10.1111/j.1365-294X.2005.02553.x 15969739

[B46] Rosenberg NA DISTRUCT: a program for the graphical display of population structure Mol Ecol Notes 2004 4 137 138 10.1046/j.1471-8286.2003.00566.x

[B47] Gao H Williamson S Bustamante CD A Markov chain Monte Carlo approach for joint inference of population structure and inbreeding rates from multilocus genotype data Genetics 2007 176 1635 1651 10.1534/genetics.107.072371 17483417 PMC1931536

[B48] Patterson N Price AL Reich D Population structure and eigenanalysis PLoS Genet 2006 2 e190 10.1371/journal.pgen.0020190 17194218 PMC1713260

[B49] Weir B Cockerham C Estimating F-statistics for the analysis of population structure Evolution 1984 38 1358 1370 10.2307/2408641 28563791

[B50] Weir BS Genetic Data Analysis II 1996 Sinauer, Sunderland, MA

[B51] Luo MC Deal KR Akhunov ED Akhunova AR Anderson OD Anderson JA Blake N Clegg MT Coleman-Derr D Conley EJ Crossman CC Dubcovsky J Gill BS Gu YQ Hadam J Heo HY Huo N Lazo G Ma Y Matthews DE McGuire PE Morrell PL Qualset CO Renfro J Tabanao D Talbert LE Tian C Toleno DM Warburton ML You FM Genome comparisons reveal a dominant mechanism of chromosome number reduction in grasses and accelerated genome evolution in Triticeae Proc Natl Acad Sci USA 2009 106 15780 15785 10.1073/pnas.0908195106 19717446 PMC2747195

[B52] Benjamini Y Hochberg Y Controlling the false discovery rate: a practical and powerful approach to multiple testing J Roy Stat Soc Ser B 1995 57 289 300

[B53] The R Project for Statistical Computing http://www.r-project.org

[B54] Gabriel SB Schaffner SF Nguyen H Moore JM Roy J Blumenstiel B Higgins J DeFelice M Lochner A Faggart M Liu-Cordero SN Rotimi C Adeyemo A Cooper R Ward R Lander ES Daly MJ Altshuler D The structure of haplotype blocks in the human genome Science 2002 296 2225 2229 10.1126/science.1069424 12029063

[B55] Barrett JC Fry B Maller J Daly MJ Haploview: analysis and visualization of LD and haplotype maps Bioinformatics 2005 21 263 265 10.1093/bioinformatics/bth457 15297300

[B56] Hamblin MT Close TC Bhat PR Chao S Kling JG Abraham KJ Blake T Brooks WS Cooper B Griffey CA Hayes P Hole D Horsley R Obert D Smith K Ullrich S Muehlbauer G Jannink JL Population structure and linkage disequilibrium in US barley germplasm: implications for association mapping Crop Science 2010 50 556 566 10.2135/cropsci2009.04.0198

[B57] Yan J Shah T Warburton ML Buckler ES McMullen MD Crouch J Genetic characterization and linkage disequilibrium estimation of a global maize collection using SNP markers PLoS One 2009 4 e8451 10.1371/journal.pone.0008451 20041112 PMC2795174

[B58] Weir BS Cardon LR Anderson AD Nielsen DM Hill WG Measures of human population structure show heterogeneity among genomic regions Genome Res 2005 15 1468 1476 10.1101/gr.4398405 16251456 PMC1310634

[B59] Börner A Korzun V Worland AJ Comparative genetic mapping of loci affecting plant height and development in cereals Euphytica 1998 100 245 248

[B60] Yan L Loukoianov A Tranquilli G Helguera M Fahima T Dubcovsky J Positional cloning of wheat vernalization gene VRN1 Proc Natl Acad Sci USA 2003 100 6263 6268 10.1073/pnas.0937399100 12730378 PMC156360

[B61] Sourdille P Snape JW Cadalen T Charmet G Nakata N Bernard S Bernard M Detection of QTLs for heading time and photoperiod response in wheat using a doubled-haploid population Genome 2000 43 487 494 10.1139/gen-43-3-487 10902713

[B62] Devos KM Dubcovsky J Dvořk J Chinoy CN Gale MD Structural evolution of wheat chromosomes 4A, 5A, and 7B and its impact on recombination Theor Appl Genet 1995 91 282 288 10.1007/BF00220890 24169776

[B63] Akbari M Wenzl P Caig V Carling J Xia L Yang S Uszynski G Mohler V Lehmensiek A Kuchel H Hayden MJ Howes N Sharp P Vaughan P Rathmell B Huttner E Kilian A Diversity arrays technology (DArT) for high-throughput profiling of the hexaploid wheat genome Theor Appl Genet 2003 113 1409 1420 10.1007/s00122-006-0365-4 17033786

[B64] White J Law JR MacKay I Chalmers KJ Smith JS Kilian A Powell W The genetic diversity of UK, US and Australian cultivars of Triticum aestivum measured by DArT markers and considered by genome Theor Appl Genet 2008 116 439 453 10.1007/s00122-007-0681-3 18060539

[B65] Yan L Helguera M Kato K Fukuyama S Sherman J Dubcovsky J Allelic variation at the *VRN-1 *promoter region in polyploid wheat Theor Appl Genet 2004 109 1677 1686 10.1007/s00122-004-1796-4 15480533

[B66] Zhang XK Xia XC Xiao YG Dubcovsky J He ZH Allelic variation at the vernalization genes *Vrn-A1*, *Vrn-B1*, *Vrn-D1 *and *Vrn-B3 *in Chinese common wheat cultivars and their association with growth habit Crop Sci 2008 48 458 470 10.2135/cropsci2007.06.0355

[B67] Islam-Faridi MN Worland AJ Law CN Inhibition of ear-emergence time and sensitivity to day-length determined by the group 6 chromosomes of wheat Heredity 1996 77 572 580 10.1038/hdy.1996.184

[B68] Noor MA Garfield DA Schaeffer SW Machado CA Divergence between the Drosophila pseudoobscura and D. persimilis genome sequences in relation to chromosomal inversions Genetics 2007 177 1417 1428 10.1534/genetics.107.070672 18039875 PMC2147956

[B69] Qi L Friebe B Gill BS Complex genome rearrangements reveal evolutionary dynamics of pericentromeric regions in the Triticeae Genome 2006 49 1628 1639 10.1139/G06-123 17426778

[B70] Dubcovsky J Dvorak J Genome plasticity a key factor in the success of polyploid wheat under domestication Science 2007 316 1862 1866 10.1126/science.1143986 17600208 PMC4737438

[B71] Villareal RL Mujeeb-Kazi A Fuentes-Davila G Rajaram S Registration of four synthetic hexaploid wheat germplasm lines derived from Triticum turgidum × T. tauscii crosses and resistant to Karnal bunt Crop Sci 1996 36 218 10.2135/cropsci1996.0011183X003600010056x

[B72] Mujeeb-Kazi A Gilchrist LI Villareal RL Delgado R Registration of 10 wheat germplasms resistant to Septoria tritici leaf blotch Crop Sci 2000 40 590 591

[B73] Warburton ML Crossa J Franco J Kazi M Trethowan R Rajaram S Pfeiffer W Zhang P Dreisigacker S van Ginkel M Bringing wild relatives back into the family: recovering genetic diversity in CIMMYT improved wheat germplasm Euphytica 2006 149 289 301 10.1007/s10681-005-9077-0

[B74] Pritchard JK Przeworski M Linkage disequilibrium in humans: models and data Am J Hum Genet 2001 69 1 14 10.1086/321275 11410837 PMC1226024

[B75] Dvorak J Luo MC Yang ZL Zhang HB The structure of the *Aegilops tauschii *genepool and the evolution of hexaploid wheat Theor Appl Genet 1988 97 657 670 10.1007/s001220050942

[B76] International HapMap Consortium A haplotype map of the human genome Nature 2005 437 1299 1320 10.1038/nature04226 16255080 PMC1880871

[B77] de Bakker PI Yelensky R Pe'er I Gabriel SB Daly MJ Altshuler D Efficiency and power in genetic association studies Nat Genet 2005 37 1217 1223 10.1038/ng1669 16244653

[B78] Aranzana MJ Kim S Zhao K Bakker E Horton M Jakob K Lister C Molitor J Shindo C Tang C Toomajian C Traw B Zheng H Bergelson J Dean C Marjoram P Nordborg M Genome-wide association mapping in Arabidopsis identifies previously known flowering time and pathogen resistance genes PLoS Genet 2005 1 e60 10.1371/journal.pgen.0010060 16292355 PMC1283159

[B79] Gore MA Chia JM Elshire RJ Sun Q Ersoz ES Hurwitz BL Peiffer JA McMullen MD Grills GS Ross-Ibarra J Ware DH Buckler ES A First-Generation Haplotype Map of Maize Science 2009 326 1115 1117 10.1126/science.1177837 19965431

[B80] Flint-Garcia SA Thuillet AC Yu J Pressoir G Romero SM Mitchell SE Doebley J Kresovich S Goodman MM Buckler ES Maize association population: a high-resolution platform for quantitative trait locus dissection Plant J 2005 44 1054 1064 10.1111/j.1365-313X.2005.02591.x 16359397

