# Supplementary figures and images for: Population- and genome-specific patterns of linkage disequilibrium and SNP variation in spring and winter wheat (Triticum aestivum L.)
Source: BMC Genomics. 2010 Dec 29;11:727. doi: 10.1186/1471-2164-11-727 (PMC3020227; doi:10.1186/1471-2164-11-727)

## Slide 1
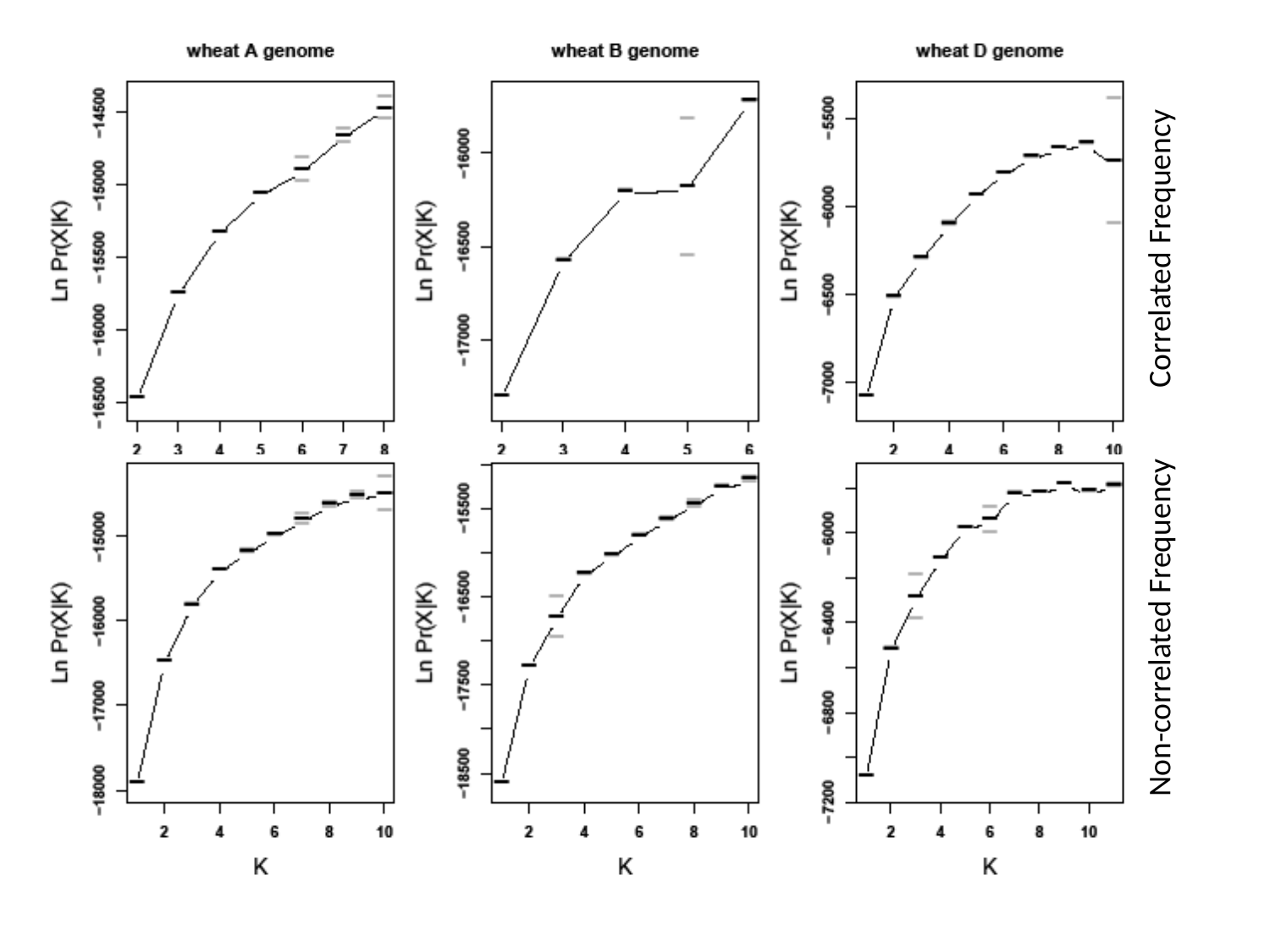

Correlated Frequency
Non-correlated Frequency

Supplement: Additional file 4 — Relationship between the log probability of data and the number of clusters K. The log probability of data (Ln Pr(X|K)) was plotted as a function of the number of clusters K for different SNP datasets and structure models assuming correlated (top three graphs) and independent (bottom three graphs) alleles frequencies. Means (black bars) and 95% confidence intervals (grey bars) of log probability of data Ln Pr(X|K) for each value of K were calculated from 10 independent runs of Structure with 100,000 burn-in steps and 106 simulation steps. [file 1471-2164-11-727-S4.PPT]

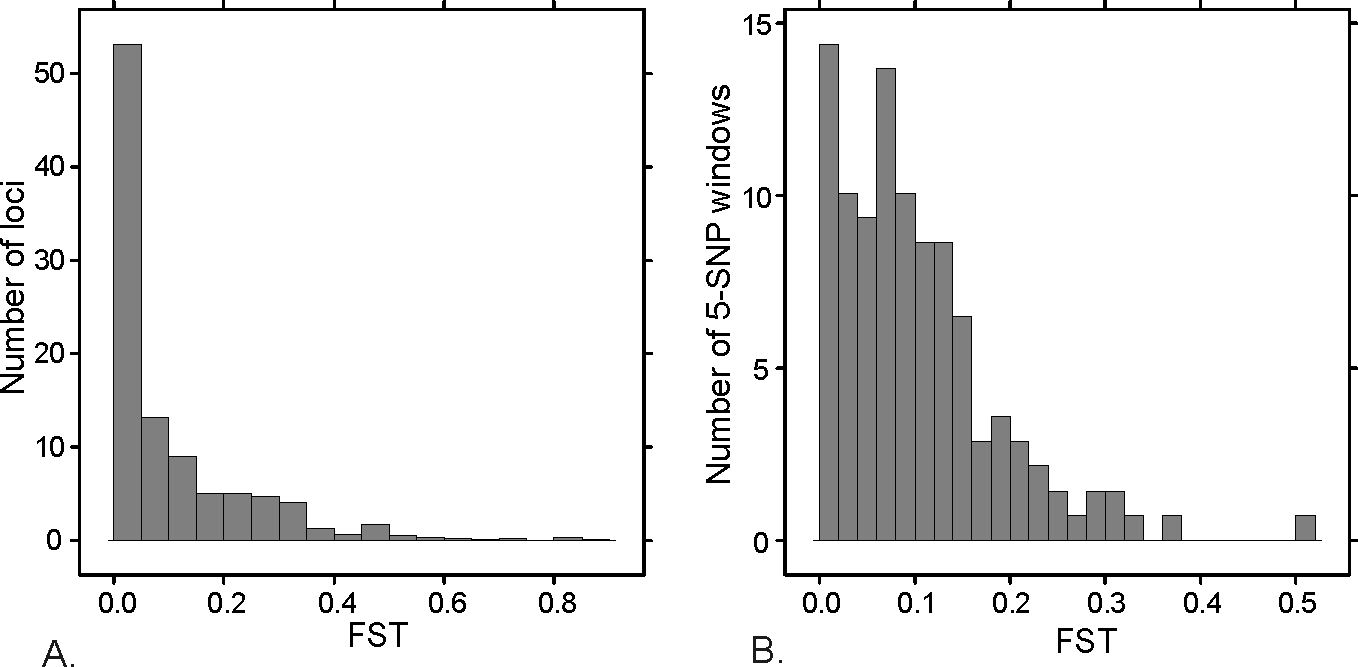

Supplement: Additional file 7 — Distribution of FST estimates for individual SNP loci and windows of 5 SNPs. A) The distribution of single-locus FST values between spring and winter wheat populations. B) The distribution of FST values in a sliding window of 5 consecutively located SNP loci. [file 1471-2164-11-727-S7.TIFF]
